# Electrochemical Immunosensor for Point-of-Care Detection of Citrullinated Histone H3 Using Screen-Printed Graphene Electrodes and a Portable Potentiostat

**DOI:** 10.1021/acsomega.6c06505

**Published:** 2026-08-19

**Authors:** Vitsarut Primpray, Wichayaporn Kamsong, Assawapong Sappat, Theeraphud Phunin, Anurat Wisitsoraat, Awirut Charoensappakit, Kritsanawan Sae-khow, Asada Leelahavanichkul, Chanpen Karuwan

**Affiliations:** † National Nanotechnology Center (NANOTEC), 61191 National Science and Technology Development Agency (NSTDA), Pathum Thani 12120, Thailand; ‡ Department of Clinical Microscopy, Faculty of Allied Health Sciences, 26683 Chulalongkorn University, Bangkok 10330, Thailand; § Center of Excellence on Translational Research in Inflammation and Immunology (CETRII), Department of Microbiology, Faculty of Medicine, Chulalongkorn University, Bangkok 10330, Thailand

## Abstract

Citrullinated histone H3 (CitH3) is an emerging biomarker for early sepsis diagnosis. However, its detection based on standard enzyme-linked immunosorbent assay (ELISA) suffers from long analysis time (4–5 h), expensive instruments, and the need for skilled operators, necessitating the development of alternative detection platforms. Herein, a portable electrochemical immunosensor for CitH3 determination was developed using a screen-printed graphene electrode coupled with a custom-designed potentiostat. CitH3 was detected via a sandwich immunoassay employing horseradish peroxidase–conjugated antibody and amperometric measurement in the presence of hydroquinone and hydrogen peroxide. Key fabrication and detection parameters were systematically optimized to achieve high analytical performance. Under optimal conditions, the immunosensor exhibited a linear detection range of 0.1–1000 ng mL^–1^ with a detection limit of 42.83 pg mL^–1^ and a quantification limit of 100.89 pg mL^–1^, enabling the discrimination between sepsis and nonsepsis conditions. Additionally, the developed method provided a lower detection limit and shorter processing time (2 h) compared with ELISA. It also demonstrated high selectivity against common serum interferents, satisfactory accuracy (recovery = 89.49–106.11%), and good repeatability (relative standard deviation < 10%). Analysis of clinical serum samples (*n* = 50) showed a high correlation coefficient with ELISA (*r* = 0.9861), and Bland–Altman analysis confirmed their good agreement. Furthermore, the assay diagnostic performance demonstrated 100% specificity with high sensitivity values of 90.01% and 81.25% for sepsis and severe sepsis cases, respectively. These results suggested the potential of the proposed portable electrochemical immunosensor as a practical tool for sepsis diagnosis.

## Graphical abstract

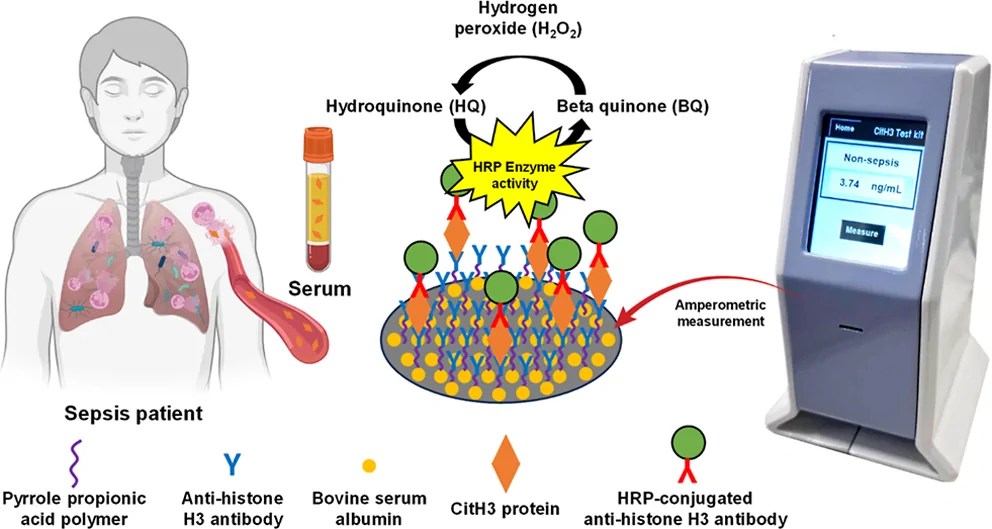

## 1 Introduction

Pneumonia is an inflammatory disease of the lungs, mostly caused by infection with a wide range of pathogenic microorganisms, including *Streptococcus pneumoniae*, *Haemophilus influenzae* type b, *Chlamydia pneumoniae*, *Legionella* spp., *Mycoplasma pneumoniae*, respiratory syncytial virus, influenza virus, and severe acute respiratory syndrome coronavirus 2. These pathogens induce pulmonary inflammation, leading to severe complications, such as sepsis and sepsis-induced acute respiratory distress syndrome, which is characterized by dysregulated host inflammatory responses and multiorgan dysfunction.^1,2^ The incidence and mortality rates of sepsis are significantly higher among elderly populations (>65 years), largely due to age-related physiological decline and impaired immunity.^3,4^ Moreover, comorbidities, such as diabetes mellitus, cancers, and immune disorders, as well as the use of immunosuppressive or corticosteroid medications, further increase both the risk and severity of sepsis in elderly patients.^5–7^ Therefore, early detection of sepsis in elderly patients is critical for timely diagnosis and effective treatment.

Several biomarkers associated with infection and sepsis, including C-reactive protein (CRP), procalcitonin (PCT), interleukin-6 (IL-6), interleukin-10, and tumor necrosis factor, are created via infection-induced systemic inflammatory responses. However, these markers are released only after the onset of inflammation (approximately 2–48 h) and generally lack specificity connecting to the source of inflammation. Consequently, their clinical utility often requires additional information, including patient history, physical examination, and other diagnostic data.^8–13^ Citrullinated histone H3 (CitH3) is a well-established biomarker of neutrophil extracellular traps (NETs). It is generated through the hydrolytic conversion of arginine and methylarginine residues in histone H3 into citrulline via peptidyl-arginine deiminase type 4.^14,15^ This post-translational modification promotes chromatin decondensation, a critical step that triggers the release of NETs. This process, known as NETosis, is an essential mechanism of the innate immune response, during which neutrophils expel nuclear components, primarily DNAs and histones, into the extracellular space to trap and neutralize invading pathogens. Serum CitH3 levels have been reported to increase as early as 0.5 h to reach a peak at 12 h, and keep increasing up to 24 h after infection. Therefore, CitH3 is a potential biomarker for the early detection of sepsis and associated disorders.^16–18^

Presently, several analytical methods for CitH3 detection have been developed based on the enzyme-linked immunosorbent assay (ELISA) principle, in which antihistone antibodies are immobilized onto microplates via streptavidin–biotin linkages to capture CitH3 from serum samples.^19–21^ Additionally, anti-CitH3 secondary antibodies are employed to specifically recognize citrullinated residues at R2, R8, and R17 of CitH3 protein, followed by detecting an absorbance-based signal derived from the enzymatic reaction between 3,3′,5,5′-tetramethylbenzidine substrate and horseradish peroxidase (HRP) conjugated antirabbit antibodies. The method exhibits a limit of detection (LOD) of approximately 5 ng mL^–1^, which is sufficient to discriminate between sepsis (101.5 ng mL^–1^) and nonsepsis patients (8 ng mL^–1^). However, the assay suffers from its long analysis time (approximately 4–5 h), expensive instruments, and the need for skilled operators.^22,23^ Thus, alternative approaches for CitH3 detection should be developed to overcome these limitations.

An electrochemical immunosensor can be a promising method for CitH3 detection owing to high specificity, high sensitivity, rapid response, portability, and ease of operation. In this method, CitH3 is detected via the electrochemical response caused by specific interactions between targeted molecules and recognition elements, such as antibodies, aptamers, enzymes, or molecularly imprinted polymers, immobilized on sensor electrode surfaces.^24–29^ However, there has still been no demonstration of an electrochemical immunosensor for CitH3 detection. Herein, a graphene-based enzymatic electrochemical immunosensor combined with a portable potentiostat for CitH3 detection was proposed for the first time. The immunosensor utilized a screen-printed graphene electrode (SPGE) fabricated from a composite of carbon paste and graphene nanoplatelets. Graphene, a two-dimensional nanocarbon material, was employed to enhance the electrical conductivity, effective surface area, and performance of a screen-printed carbon electrode (SPCE).^30–32^ To enable efficient biorecognition, the surface of SPGE was modified with poly­(pyrrolepropionic acid), a conductive polymer enriched with carboxyl functional groups that allowed stable covalent immobilization of anticitrullinated histone H3 (citrulline R2, R8, R17, and R26) antibodies. In the detection process, CitH3 would specifically bind to the immobilized antibodies on SPGE surfaces. Subsequently, HRP-conjugated antihistone H3 antibodies were additionally introduced to form sandwich immunocomplexes. The CitH3 concentration was determined from the electrochemical current generated due to the HRP redox reaction. Furthermore, a customized portable potentiostat was developed to enable on-site signal readout and support point-of-care (POC) sepsis diagnosis.

## 2 Experimental Section

### 2.1 Reagents and Materials

Carbon paste (item code: EIC CR 25) was purchased from Elemental Inks & Chemicals (New Jersey, USA). Silver/silver chloride paste (item code: C61003P7) and insulating paste (item code: D2070423P5) were acquired from Sun Chemical (New Jersey, USA). Graphene nanoplatelet powder was obtained from UC Bacon (Taoyuan City, Taiwan). 1H-pyrrole-1-propionic acid (PPA) (≥97%), 2-(*N*-morpholino) ethanesulfonic acid (MES) hydrate (≥99%), 1-ethyl-3-(3-(dimethylamino)­propyl) carbodiimide hydrochloride (EDC, ≥99%), *N*-hydroxysuccinimide (NHS, ≥98%), bovine serum albumin (BSA), and hydroquinone (≥99%) were bought from Sigma-Aldrich (St. Louis, MO, USA). Phosphate-buffered saline (PBS, pH 7.2) powder was ordered from Thermo Fisher Scientific (Rockford, IL, USA). Anticitrullinated histone H3 (citrulline R2 + R8 + R17) antibodies (cat no.: ab5103), Anticitrullinated histone H3 (citrulline R26) antibodies (cat no.: ab212082), and HRP-conjugated antihistone H3 antibodies (cat no.: ab21054) were gotten from abcam (Cambridge, UK). Polyethylene terephthalate (PET) sheets with a nominal thickness of 250 μm were procured from Victor & R Company Limited (Bangkok, Thailand). Aluminum block screens and stainless-steel screen masks with electrode patterns were supplied by Mitani Micronics (Thailand) Company Limited.

### 2.2 Fabrication of CitH3 Immunosensors

The conductive ink was prepared by incorporating 5% (w/w) graphene nanoplatelets into carbon paste, followed by homogenization via ball milling (Retsch Emax, Germany) at 1000 rpm for 60 min.^24^ Next, the conductive ink was screen-printed on PET substrates via a designed stainless-steel screen mask to fabricate working electrodes (WE) and counter electrodes (CE) using a screen printer (DEK model 03ix, USA). Silver/silver chloride paste was then screen-printed via another screen mask to form a reference electrode. After each printing step, the electrodes were dried at 60 °C for 15 min. Finally, an insulating paste was screen-printed via the other screen mask to define the active electrode areas and then cured at 90 °C for 15 min. The resulting SPGE exhibited a macroscopic working area of 12.57 mm^2^ and a total length of 25 mm.

Subsequently, the electrode was modified with carboxylic groups by electrochemical polymerization using 100 μL of 7.5 mM PPA in 0.5 M KCl via cyclic voltammetry (CV, 10 cycles from 0.0 to +0.85 V at a scan rate of 100 mV s^–1^). The electrode was then rinsed with deionized water and dried in air at room temperature. Next, the carboxylated SPGE was activated by applying 10 μL of a 0.1 M EDC/NHS solution prepared in 25 mM MES buffer (pH 5.0) and incubating for 30 min at 25 °C to generate reactive NHS esters on the surface. Excessive activating agents were removed by rinsing with the MES buffer. For biorecognition-molecule covalent immobilization, 10 μL of a mixture containing (citrulline R2 + R8 + R17) and (citrulline R26) anticitrullinated histone H3 antibodies (10 μg mL^–1^) in the MES buffer was applied to the electrode surface and incubated for 30 min at 25 °C. The resulting CitH3 antibody-modified electrode was then washed with the MES buffer to remove unbound biomolecules. Nonspecific binding sites were blocked by dropping 10 μL of 0.5% (w/v) BSA in the MES buffer and incubating for 30 min at 25 °C. Finally, the electrode was rinsed with 10 mM PBS buffer (pH 7.4) and dried in air (Figure 1A).

**Figure 1:**
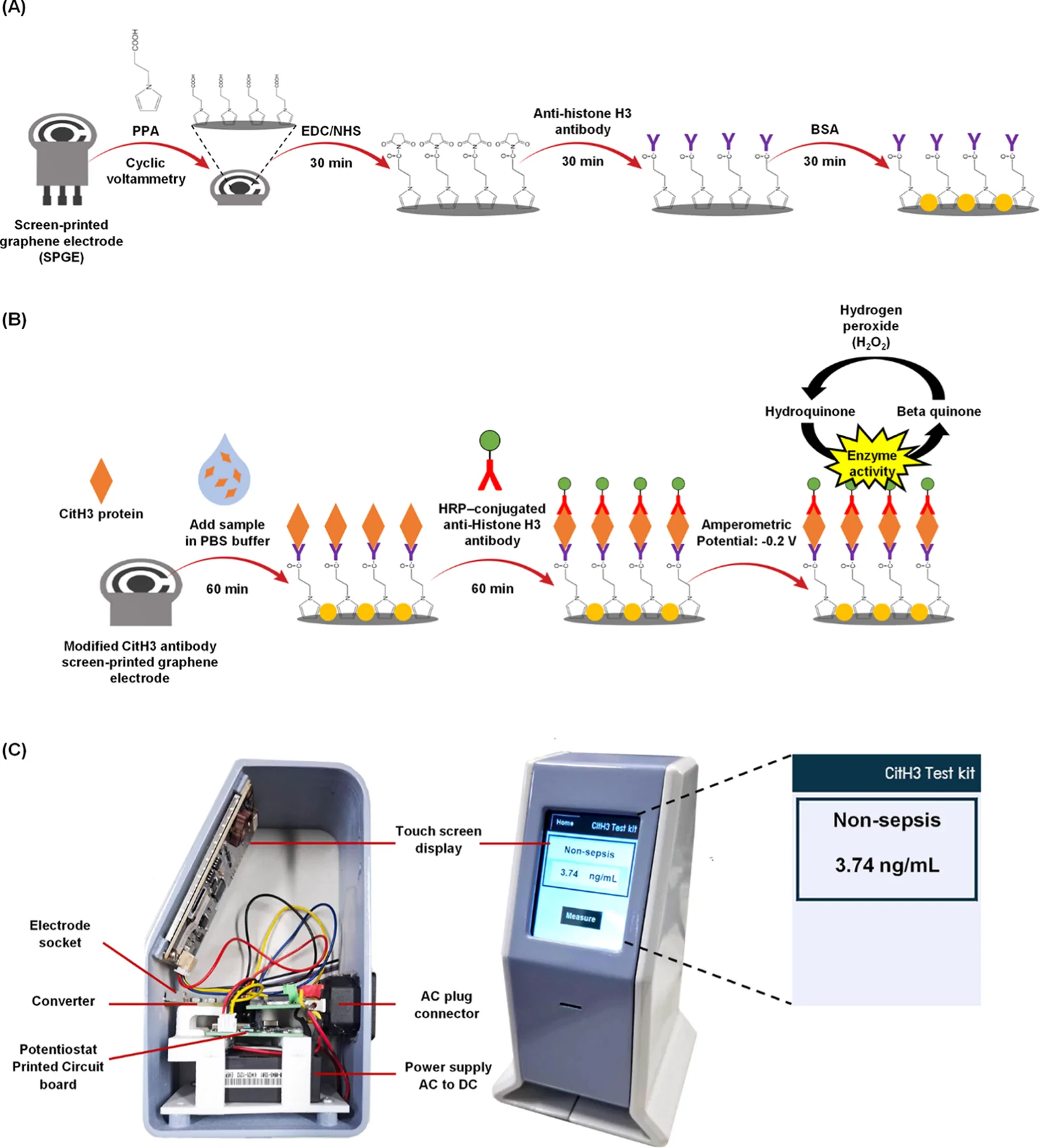
Schematic illustration of the immunosensor coupled with the customized portable potentiostat: (A) preparation of a modified SPGE, (B) electrochemical detection procedure, and (C) design of the portable potentiostat.

### 2.3 Electrochemical Detection

Figure 1B schematically presents the CitH3 electrochemical detection procedure using the developed immunosensor. First, 10 μL of a CitH3 sample was mixed with 90 μL of PBS buffer (10 mM, pH 7.4). Subsequently, 10 μL of the sample mixture was dispensed onto the WE and incubated at 25 °C for 60 min, allowing CitH3 to specifically bind to the antibodies immobilized on the WE surface. The electrode was then gently rinsed with PBS (10 mM, pH 7.4) to remove unbound proteins. Next, 10 μL of HRP-conjugated antihistone H3 antibodies (7.5 μg mL^–1^) was applied to the WE and incubated at 25 °C for 60 min to bind with captured CitH3 and form sandwich immunocomplexes. Unbound antibodies were then removed by washing with PBS (10 mM, pH 7.4) containing 1% (w/v) BSA. Subsequently, 45 μL of hydroquinone (1 mM) in 50 mM PBS (pH 7.4) as a redox mediator was dropped onto the WE, followed by adding 5 μL of hydrogen peroxide (50 mM) in 50 mM PBS (pH 7.4) as the enzymatic substrate (Figure 1B). Finally, the amperometric current was recorded continuously up to 200 s for the quantitative determination of the CitH3 concentration.

### 2.4 Electrochemical Measurements

CV was carried out to evaluate the current response of 5 mM K_3_Fe­(CN)_6_ in PBS (10 mM, pH 7.4). CV measurements were conducted over a potential range of −0.3 to +0.6 V at a scan rate of 50 mV s^–1^ to study the effect of antibody immobilization. Amperometric measurements were performed at an applied potential of −0.2 V (vs Ag/AgCl) for 200 s to evaluate the enzymatic response of the HRP-conjugated protein and to optimize the blocking and detection conditions. The experiments for each condition were carried out using three independently prepared electrodes (*n* = 3).

### 2.5 Characterization

Field-emission scanning electron microscopy (Hitachi SU-8230) and Fourier-transform infrared spectroscopy (FTIR, PerkinElmer Scientific, Spectrum Two FT-IR Spectrometer) were employed to evaluate the surface morphology and chemical functional groups of the modified electrode, respectively. In addition, the fabricated immunosensors were characterized after each processing step using CV and electrochemical impedance spectroscopy (EIS) with 5 mM K_3_Fe­(CN)_6_ in PBS buffer. CV measurements were carried out over a potential range of −0.3 to +0.6 V at a scan rate of 50 mV s^–1^ to monitor changes in anodic and cathodic current responses during surface modification. Electron transfer rates were evaluated from the peak-to-peak potential separation (Δ*E*_p_), defined as Δ*E*_p_ = *E*_pa_ – *E*_pc_, where *E*_pa_ and *E*_pc_ were the anodic and cathodic peak potentials, respectively. EIS measurements were conducted over a frequency range of 10 mHz to 50 kHz with an alternating current (AC) amplitude of 10 mV. The impedance data were presented as EIS (Nyquist) plots, which were used to calculate a charge transfer resistance (*R*_ct_).

### 2.6 Construction of a Customized Portable Potentiostat

The potentiostat was mechanically designed to exhibit a vertically elongated trapezoidal shape with an overall dimension of 62 × 95 × 180 mm^3^, featuring a sloped touch screen display to improve readability and an upper front electrode socket for sensor insertion. The device enclosure was fabricated by three-dimensional printing using acrylonitrile butadiene styrene filament (Ender-7, Creality), selected owing to its low cost and mechanical robustness. The printing parameters included a nozzle temperature of 190 °C, a base plate temperature of 65 °C, and a printing speed of 250 mm s^–1^. The potentiostat operated using an AC power source, which was converted to direct current (DC) via a power supply module. Then, the DC power supply voltage was adjusted using a voltage converter to an appropriate level for driving the ESP32 microcontroller on the main potentiostat printed circuit board. The microcontroller coordinated system operation and data acquisition while interfacing with a digital-to-analog converter (MCP4728) to generate a precise voltage signal. The signal was applied to the inserted electrodes to generate a current response. The resulting current was amplified using an operational amplifier (TLC2264) and transmitted to a 24 bit analog-to-digital converter (ADS1256), enabling accurate measurements of low-level current signals. The digitized data were then sent to the microcontroller for processing and analysis.

In a detection step, the developed device potentiostat operated in an amperometric mode by applying a constant potential of −0.2 V (vs Ag/AgCl) and recording the resulting current response, which would be shown in real time on the touch screen display. To perform a measurement, the electrodes were inserted into the socket, followed by the addition of the hydroquinone reagent and H_2_O_2_ solution. When the Measure command on the touch screen was activated, the potential of −0.2 V was applied for 200 s while recording the amperometric current signal. The obtained current response at 200 s was quantified using a predetermined calibration curve to determine the CitH3 concentration, which would then be displayed on the screen. When the concentration was lower than 0.1 ng mL^–1^, the result of “negative” would be shown without a numerical value, indicating that the concentration was lower than the quantification limit of the sensor. For the concentrations between 0.1 and 50 ng mL^–1^, the result would be classified and displayed as “non-sepsis” presented together with the corresponding numerical concentration. When the concentration ranged from 50 to 83 ng mL^–1^, the result of “sepsis” would be identified and shown with the numerical value. For concentrations exceeding 83 ng mL^–1^, the result of “severe sepsis” would be reported along with the numerical concentration. The concentration thresholds for the classification were established based on clinical evaluation involving the association between physician assessments and ELISA measurements.

### 2.7 Real Sample Analysis and Comparison

To evaluate the practical applicability of the proposed immunosensor, human serum samples (*n* = 50) were collected for clinical evaluation under the protocols approved by the Institutional Review Board of Faculty of Medicine, Chulalongkorn University, Bangkok, Thailand (COA no. 1232/2024). The samples were classified by physicians according to the Third International Consensus Definitions for Sepsis and Septic Shock (Sepsis-3).^33^ Specifically, the concentration thresholds for the nonsepsis, sepsis, and severe sepsis groups were assigned to provide optimal discrimination among our clinical cohort. They were analyzed under the optimized experimental conditions. The serum samples were tested directly using the developed electrochemical immunosensor coupled with the customized portable potentiostat. The obtained amperometric current responses were converted to CitH3 concentrations using an embedded calibration curve. The analytical performance of the proposed method was validated by comparing it with a commercial CitH3 ELISA kit as the standard reference method. Correlation analysis was performed to determine the relationship between the two methods, and a Bland–Altman plot was constructed to assess the agreement and systematic bias. The clinical sensitivity and specificity for identifying a sepsis condition by the developed method were calculated from the definitions: sensitivity = 100% × TP/(TP + FN) and specificity = 100% × TN/(TN + FP), where TP = true positive, FN = false negative, TN = true negative, and FN = false negative referenced to ELISA.

## 3 Results and Discussion

### 3.1 Optimization of the Immunosensor

To achieve specific CitH3 detection, the CitH3 antibodies were immobilized on the WE with different concentrations and incubation times. The optimal immobilization conditions were determined by evaluating the CV response to 5 mM K_3_Fe­(CN)_6_ in PBS buffer (Figure 2). From Figure 2A, the current response decreased from 48.65 ± 0.30 μA to 19.16 ± 1.01 μA as the CitH3 antibody concentration increased from 0 to 10 μg mL^–1^ (incubation time = 60 min). The decreased oxidation current could be ascribed to the impeded electron transfer caused by the formation of an insulating immobilized antibody layer on the WE surface. At higher antibody concentrations, the current response remained nearly constant, indicating the saturation of the electrode surface and suggesting an optimal CitH3 antibody concentration of 10 μg mL^–1^. Additionally, the effect of immobilization time was investigated in the range of 0–60 min at the optimal CitH3 antibody concentration, as displayed in Figure 2B. It could be seen that the current response decreased from 46.04 ± 0.92 μA to 20.78 ± 1.63 μA with increasing incubation time from 0 to 30 min. Prolonged incubation time of more than 30 min did not result in a significant additional change in the current response, implying a full surface coverage of antibodies. Therefore, the antibody concentration of 10 μg mL^–1^ and the incubation time of 30 min were considered suitable conditions for CitH3 antibody immobilization.

**Figure 2:**
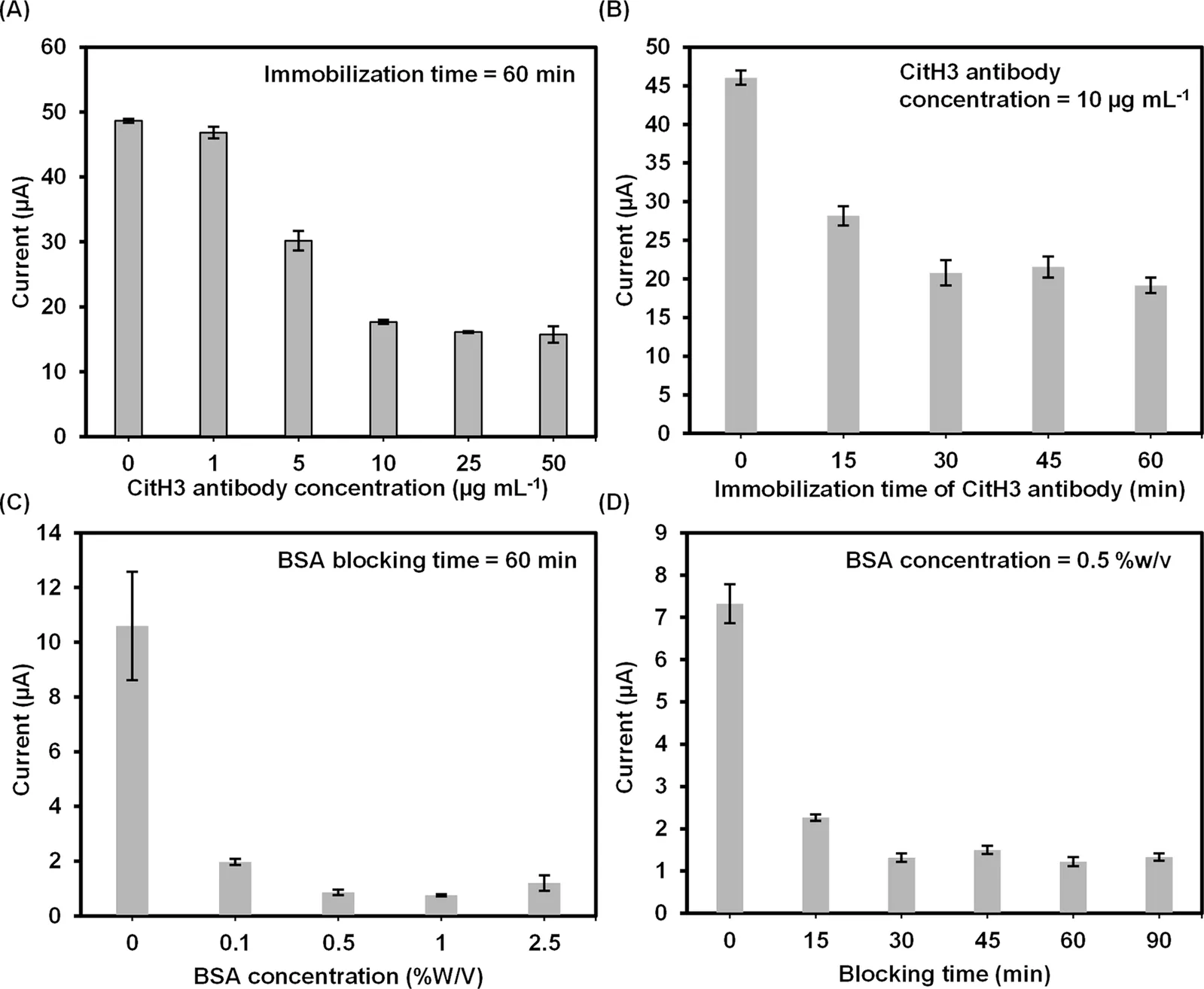
Optimization of immobilization parameters: (A) CitH3 antibody concentration and (B) immobilization time evaluated by the oxidation current of 5 mM K_3_Fe­(CN)_6_ and blocking conditions: (C) BSA concentration and (D) blocking time assessed from the nonspecific enzymatic current response.

Generally, an electrode surface was susceptible to nonspecific adsorption of other molecules that could be present in the sample. To minimize this effect, BSA was employed to prevent nonspecific binding. The BSA blocking conditions were optimized by varying BSA concentrations and incubation times. The blocking efficiency was evaluated by measuring the enzymatic amperometric current response to HRP–conjugated proteins in the absence of CitH3. As shown in Figure 2C,D, the current responses decreased with increasing BSA concentration and incubation time before slightly rising at high BSA concentrations and long incubation times, resulting in the minimum values of 0.86 ± 0.10 μA at 0.5% (w/w) BSA and 1.25 ± 0.09 μA at an incubation time of 30 min. Thus, 0.5% (w/w) BSA and 30 min incubation time were selected as the optimal nonspecific binding conditions of the immunosensor.

### 3.2 Characteristics of the Modified Screen-Printed Electrodes

The morphologies and surface functional groups of the fabricated biosensors were evaluated using SEM and FTIR. Figure 3A presents the typical surface of a SPGE consisting of densely packed carbon nanoparticles distributed around some wrinkled graphene sheets. The graphene flakes displayed polygonal shapes with varying dimensions from a few hundred nanometers to a few microns, while the carbon nanoparticles exhibited small diameters of less than 50 nm. Figure 3B reports FTIR spectra of bare SPCE, unmodified SPGE, PPA-modified SPGE (SPGE/PPA), anti-CitH3 antibody immobilized SPGE (SPGE/PPA/CitH3 ab), and BSA-blocked SPGE (SPGE/PPA/CitH3 ab/BSA). It showed that the bare SPCE and SPGE displayed insignificant signals of functional groups. After PPA modification, SPGE/PPA exhibited characteristic absorption bands in the ranges of 1000–1050 cm^–1^, 1500–1650 cm^–1^, and 2850–2950 cm^–1^ corresponding to C–O, amide I&II groups, and C–H of PPA, respectively. Following the anti-CitH3 antibody immobilization (SPGE/PPA/CitH3 ab), the additional broad band at 1050–1250 cm^–1^ relating to amide III pronouncedly appeared while the intensities of other bands also increased. Moreover, a broad absorption feature in the range of 3200–3600 cm^–1^, attributed to N–H stretching vibrations of protein molecules, was additionally observed. These changes signified the successful attachment of protein molecules onto the electrode surface. After BSA blocking (SPGE/PPA/CitH3 ab/BSA), the protein-related absorption bands, particularly the band around 3200–3600 cm^–1^, were enhanced, suggesting the creation of the antibody/BSA recognition layer. The progressive spectral changes observed after each modification step demonstrated the successful formation of the SPGE/PPA/CitH3 Ab/BSA biosensing interfaces.

**Figure 3:**
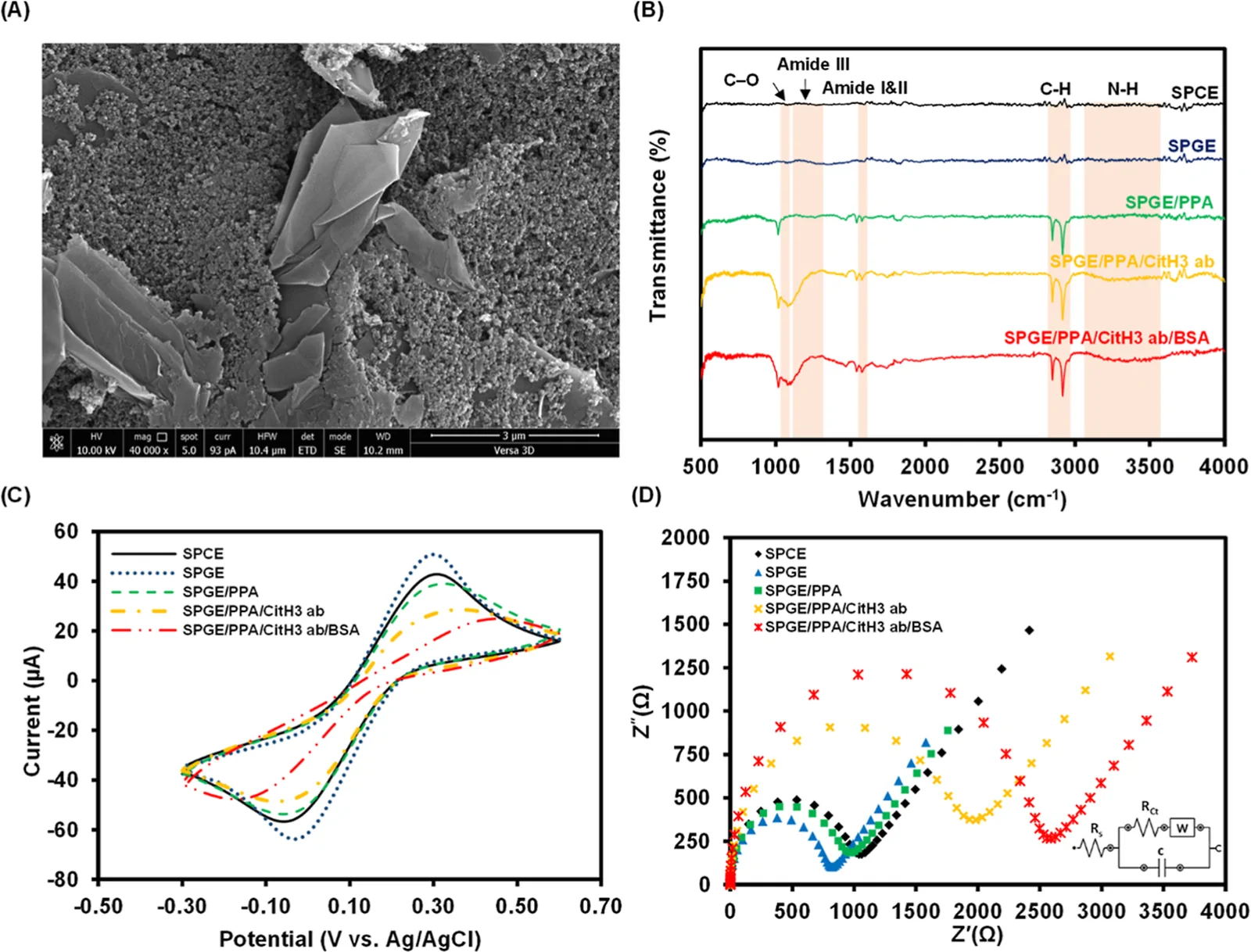
(A) SEM image of SPGE, (B) FTIR spectra, (C) cyclic voltammograms, and (D) EIS plots of SPCE, SPGE, SPGE/PPA, SPGE/PPA/CitH3, and SPGE/PPA/CitH3/BSA. CV and EIS measurements were performed in 5 mM K_3_Fe (CN)_6_, 0.1 M PBS buffer solution (pH = 7.4).

The fabricated CitH3 immunosensor was electrochemically evaluated by determining the current response and charge transfer resistance via CV and EIS after each processing step, including PPA-modification (SPGE/PPA), CitH3 antibody immobilization (SPGE/PPA/CitH3 ab), and BSA blocking (SPGE/PPA/CitH3 ab/BSA), compared with SPCE and SPGE, as shown in Figure 3C,D. From Figure 3C, SPGE exhibited higher anodic and cathodic peak currents (52.25 ± 0.12 μA and 47.77 ± 0.67 μA, respectively) with a smaller Δ*E*_p_ of 0.30 V compared with SPCE (41.77 ± 0.81 μA and 37.13 ± 0.34 μA, Δ*E*_p_ = 0.37 V). The lower Δ*E*_p_ of the SPGE indicated faster electron transfer kinetics, which could be attributed to the high surface area and superior conductivity of graphene.^24,34^ According to our previous study, the addition of 5% (w/w) graphene nanoplatelets enhanced the enzymatic current response to HRP of an electrochemical immunosensor for COVID-19 diagnosis by 31.6% compared with SPCE, demonstrating the enhancement of electron transfer between the electrode surface and electroactive species by graphene.^24^ The enhancement could directly be inferred to the present electrochemical enzymatic immunosensors, which also involved the catalytic current generated by the HRP-mediated redox reaction.

Following surface modification, the SPGE/PPA electrode exhibited anodic and cathodic peak currents of 48.77 ± 0.31 μA and 43.52 ± 0.61 μA (Δ*E*_p_ = 0.39 V), respectively. It showed that the peak currents decreased and Δ*E*_p_ increased slightly upon functionalization with PPA. After immobilizing CitH3 antibodies, the corresponding peak currents further decreased to 20.78 ± 0.16 μA and 16.21 ± 0.55 μA (Δ*E*_p_ = 0.45 V). Upon BSA blocking, the anodic and cathodic peak currents were additionally reduced to 16.43 ± 0.25 μA and 12.79 ± 0.43 μA, with a corresponding increase in Δ*E*_p_ to 0.62 V. Besides, the success of each biosensor fabrication step could be analyzed by calculating *R*_ct_ from the EIS plots (Figure 3D). From the EIS calculation, the *R*_ct_ value decreased from 1062 ± 35 Ω (SPCE) to 819 ± 26 Ω (SPGE) after graphene addition, revealing around a 25% enhancement in the charge transfer rate in accordance with our previous study.^24^ In contrast, the *R*_ct_ values increased to 1017 ± 20, 1093 ± 59, and 1377 ± 25 Ω after probe immobilization, blocking, and hybridization, respectively.

These results indicated an increase in interfacial resistance and a decrease in electron transfer at the electrode surface after each modification step due to the possible formation of semi-insulating layers involving PPA with carboxylic groups as well as biomolecules on the electrode surface.^35^ Progressive increments in *R*_ct_ through modification steps could also confirm the successful fabrication of an immunosensor. In particular, CV and EIS results showed a modest decrease in the current response and a corresponding increase in *R*_ct_ after PPA modification, indicating a moderate reduction in conductivity. Thus, the electron transfer capability remained sufficient for effective CitH3 sensing, and PPA could provide the essential surface functionalization for covalent antibody immobilization with an acceptably small adverse impact on electrochemical performance (to be further demonstrated in subsequent sections).

### 3.3 Optimization of Electrochemical Detection

To obtain an optimal sensitivity for CitH3 detection, the time for CitH3 binding with immobilized antibodies (first binding), the concentration of HRP-conjugated antihistone H3 antibodies, and the time for HRP-conjugated antibodies binding with CitH3 (second binding) were systematically varied to maximize the enzymatic current response to 10 ng mL^–1^ CitH3 (Figure 4). As shown in Figure 4A, the current response increased with increasing the first binding time, reaching a maximum value of 4.03 ± 0.02 μA at 60 min, before declining at the longest time of 90 min. For the effect of the HRP-conjugated antibody concentration as demonstrated in Figure 4B, the amperometric signal increased with rising the concentration, attaining the highest level of 4.18 ± 0.16 μA at 7.5 μg mL^–1^. In the case of the second binding time as presented in Figure 4C, the current response increased to the highest value of 3.93 ± 0.12 μA with increasing the incubation time up to 60 min, after which no further enhancement was observed. Hence, the optimal detection conditions included the first binding time of 60 min, the HRP-conjugated antibody concentration of 7.5 μg mL^–1^, and the second binding time of 60 min.

**Figure 4:**
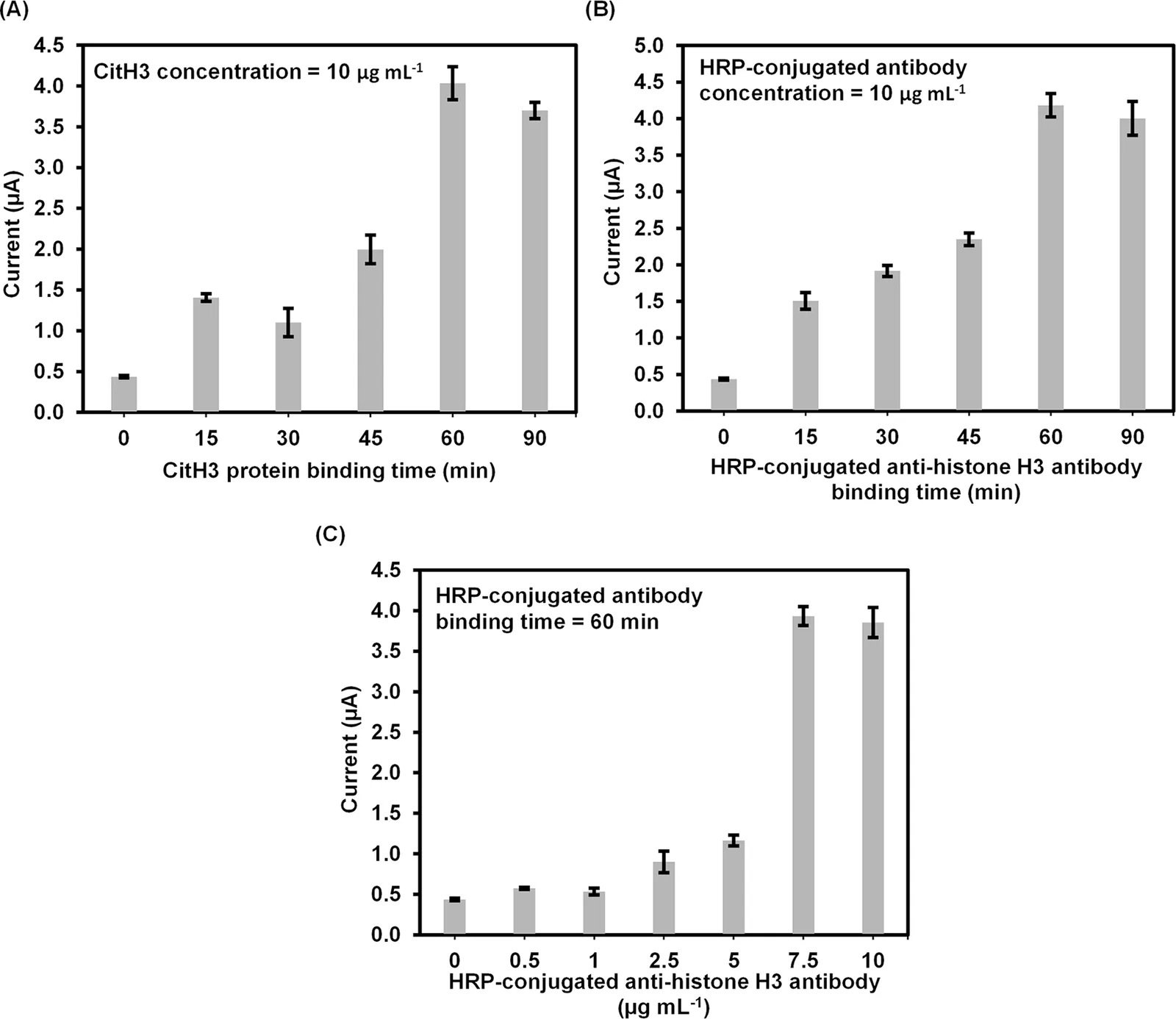
Optimization of electrochemical detection parameters: (A) the time for CitH3 binding with immobilized antibodies, (B) HRP-conjugated antihistone H3 antibody concentration, and (C) the time for HRP-conjugated antibodies binding with CitH3, evaluated from the enzymatic current response to 10 ng mL^–1^ CitH3.

### 3.4 Analytical Performance

The analytical performance of the immunosensor for CitH3 detection was systematically evaluated under optimized conditions via amperometric measurements using the developed portable potentiostat and a commercial potentiostat. As shown in Figure 5A,B, each platform displayed a concentration-dependent increase in current response with increasing CitH3 level, which could be attributed to the formation of the sandwich immunocomplexes and the associated HRP-catalyzed electrochemical reaction at the electrode surface. However, the commercial potentiostat demonstrated lower background current, superior signal stability, and higher resolution compared with the portable device, as illustrated in Figure 5C. Specifically, the background current and variability of the commercial system [1.4838 ± 0.014803 μA, % relative standard deviation (RSD) = 0.99 (*n* = 5), 5-digit resolution] were less than those of the developed device [1.691 ± 0.02954 μA, % RSD = 1.74 (*n* = 5), 4-digit resolution]. The inferior signal precision at a low current was caused by the relatively high electronic noise level and limited resolution of the portable potentiostat.

**Figure 5:**
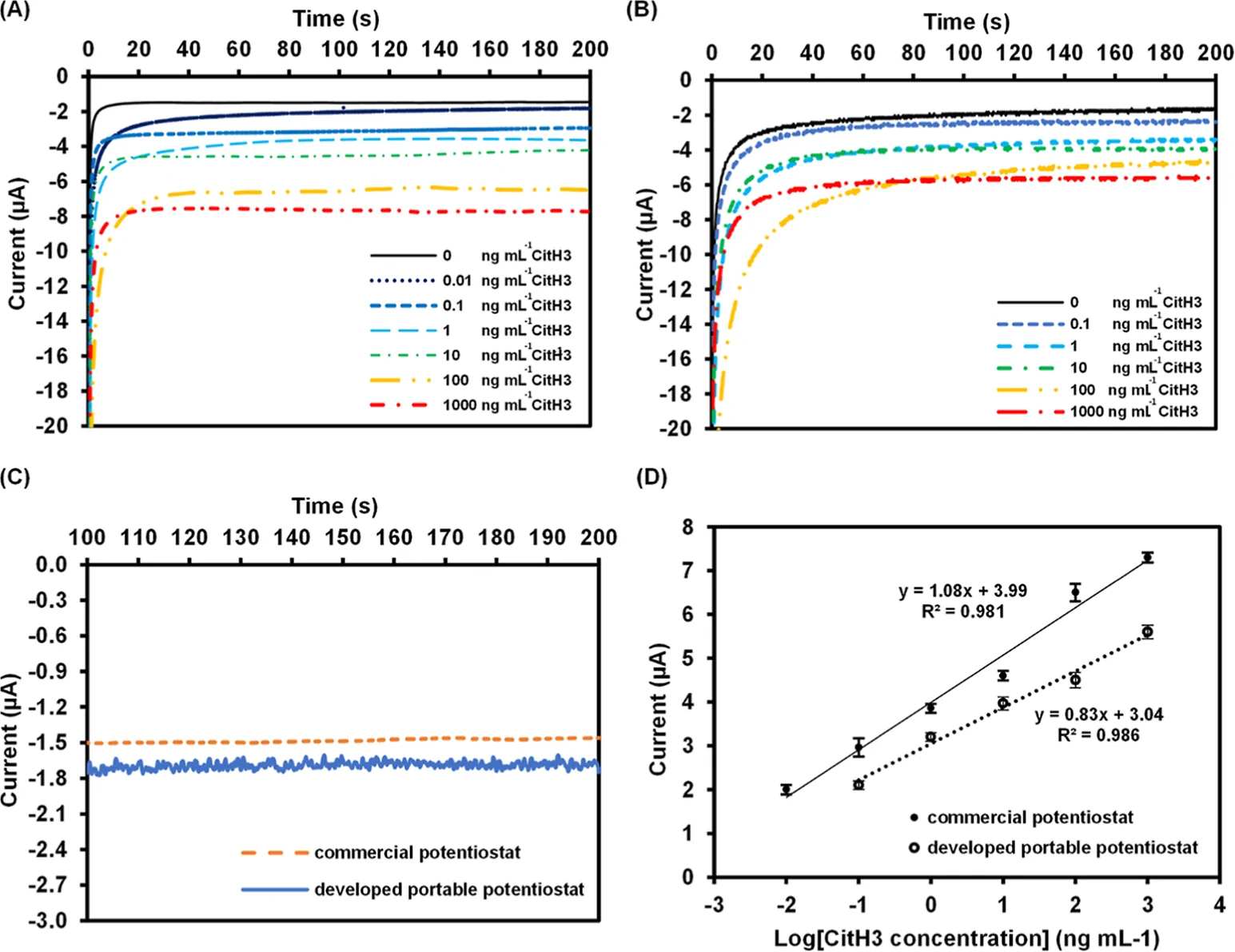
Electrochemical detection performance of CitH3 using the optimized immunosensor: amperometric responses obtained at varying CitH3 concentrations measured with (A) a commercial potentiostat and (B) the developed portable potentiostat. (C) Comparison of background noise and (D) calibration curves between the two platforms.

Calibration curves of both devices were constructed to compare their analytical characteristics, as reported in Figure 5D. Notably, the developed device provided slightly lower current values than the commercial one. Correspondingly, the portable device exhibited a linear sensitivity of 0.83 μA·mL ng^–1^ in the CitH3 concentration range of 0.1–1000 ng mL^–1^, while the commercial potentiostat gave a relatively high sensitivity of 1.08 μA·mL ng^–1^ over a wider detection range of 0.01–1000 ng mL^–1^. The narrower range of the portable device was due to its inability to reliably detect CitH3 at the lowest concentration of 0.01 ng mL^–1^. The LOD and limit of quantification (LOQ) were then calculated from the mean signal blank (*X̅*) and standard deviation (σ) as *X̅* + 3.3σ and *X̅* + 10σ, respectively. From the calculation, the portable device has an LOD of 42.83 pg mL^–1^ and an LOQ of 100.89 pg mL^–1^ compared with the values of 9.19 pg mL^–1^ and 21.34 pg mL^–1^ for the commercial instrument. The slightly inferior analytical performance of the portable device could be due to its low bias stability, high background noise level, and limited measurement resolution. The performance of the portable device could be improved in the future by employing electromagnetic shielding on the device housing and implementing digital filtering algorithms on the microcontroller to reduce background noise and enhance signal resolution.

Nevertheless, the developed device offered markedly lower LOD than that of conventional ELISA (≈5 ng mL^–1^). The achieved LOD was sufficiently low for discrimination between sepsis patients and nonsepsis individuals (<50 ng mL^–1^). In addition, the device gave advantages in portability, low cost, operational simplicity, and short processing time (2 h), which was significantly shorter than that of a typical conventional ELISA procedure (4–5 h). Additionally, the portable system allowed on-site assessment, providing both qualitative (positive/negative) and quantitative (CitH3 concentration) results. These features rendered its practical applicability to POC sepsis diagnosis.

### 3.5 Selectivity, Stability, Accuracy, and Precision

The selectivity of the fabricated CitH3 electrochemical immunosensor was evaluated by taking its response in the presence of other typical serum components, including 50 mg mL^–1^ albumin, 1 mg mL^–1^ glucose, and 3 mg mL^–1^ hemoglobin, compared with 100 ng mL^–1^ CitH3 (positive control). The measured current signal was compared with the negative control and expressed as a relative response defined as the percentage ratio between the current with a potential interferent and that of the negative control, as presented in Figure 6A. The relative responses for CitH3, the negative control, albumin, glucose, and hemoglobin were 314.88 ± 2.38%, 100.00 ± 1.66%, 100.53 ± 1.89%, 105.87 ± 1.82%, and 105.45 ± 1.58%, respectively. The values obtained for other substances were in the range of 100 ± 10%, indicating that these substances did not significantly affect the current response (low interference). Additionally, the high relative response of the positive control demonstrated that the developed immunosensor exhibited high CitH3 selectivity.

**Figure 6:**
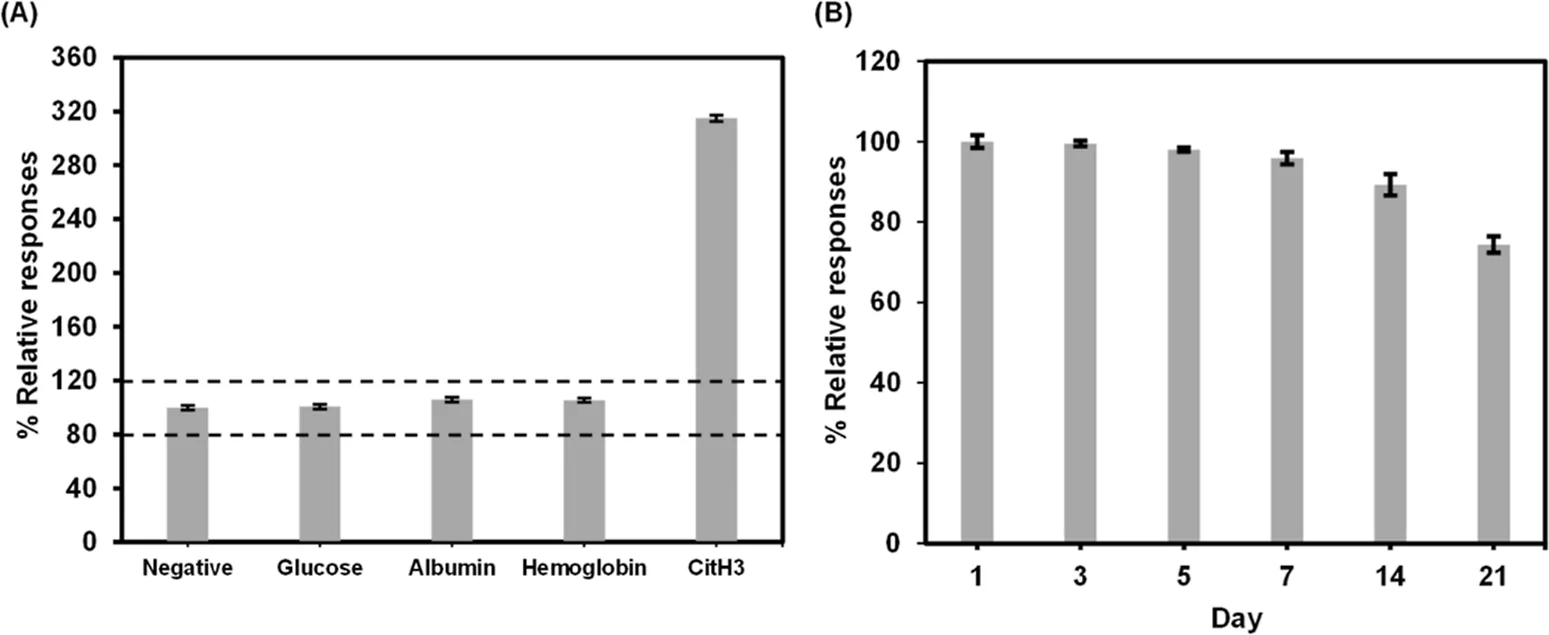
(A) Selectivity of the CitH3 immunosensor. Relative current responses in the presence of potential interferents (albumin, glucose, and hemoglobin) compared with the negative control and positive control. (B) Storage stability of the CitH3 immunosensor for 21 days under storage at 4 °C.

Additionally, the storage stability of the biosensor was assessed with 100 mg mL^–1^ CitH3 under storage at 4 °C up to 21 days. The results were presented as a relative response with respect to the value obtained on the first day, as displayed in Figure 6B. It showed that the relative response gradually declined to 89.3 ± 2.7% as the storage time increased to 14 days, presenting good storage stability. However, the relative response substantially dropped to 74.36 ± 2.04% by day 21. The behavior could be associated with the natural degradation of the immobilized biomolecules on the electrode surface during storage. Based on the results, the shelf life of the biosensor that still provided acceptable analytical performance under storage at 4 °C was around 14 days. The shelf life might be extended for practical POC applications through protective polymer coatings, optimized packaging, and dry-storage strategies in the future.

The practical applicability of the developed immunosensor for CitH3 detection in human serum samples was further evaluated using serum diluted 10-fold with PBS before analysis. CitH3 solutions were spiked into the diluted serum to assess the analytical performance at three concentrations, including 0.1 ng mL^–1^, 10 ng mL^–1^, and 1000 ng mL^–1^. Response at each concentration was measured five times using independently prepared electrodes to assess the accuracy and precision by calculating the % recovery and % RSD, respectively. From Table 1, % recovery and % RSD at the tested concentrations were in the ranges of 89.49–106.11% and 6.78–8.43%, respectively. These values fell within the acceptable limits specified by AOAC guidelines, demonstrating that the developed electrochemical immunosensor exhibited high accuracy and precision for CitH3 detection in real serum samples.

**Table 1:** Determination of CitH3 Concentrations in Human Serum Using the Developed Electrochemical Immunosensor^a^

CitH3 concentration (ng mL^–1^)	Determined concentration (ng mL^–1^)	% recovery	% RSD
0	ND		
0.1	0.11 ± 0.01	106.11	8.43
10	8.95 ± 0.68	89.49	6.78
1000	1002 ± 72	100.20	7.24

a ND = not detected.

Moreover, the intraday repeatability, interday stability, and device-to-device variation of the manufactured portable potentiostats were evaluated to verify their reliability for practical usage, as summarized in Table 2. Intraday repeatability was assessed by operating the device every 2 h over a 10 h period, whereas interday stability was determined over seven consecutive days. Device-to-device variation was assessed using three independently manufactured potentiostats with spiked serum samples at three different CitH3 concentrations (0.1, 10, and 1000 ng mL^–1^). From Table 2, the recovery and RSD values for all measurements were in the ranges of 89–104% and 6–9%, staying within the allowed limits under AOAC guidelines. Thus, the developed platform with the portable potentiostat exhibited excellent repeatability, reproducibility, and reliability suitable for quantitative CitH3 detection in human serum.

**Table 2:** Intraday Repeatability, Interday Stability, and Device-to-Device Variation of Three Independently Manufactured Potentiostats for CitH3 Detection in Human Serum

	intraday repeatability		interday stability		device-to-device variation	
CitH3 concentration (ng mL^–1^)	% recovery	% RSD	% recovery	% RSD	% recovery	% RSD
0.1	102.11	8.43	100.21	7.93	98.20	8.63
10	89.49	6.78	99.32	6.59	96.92	6.44
1000	100.20	7.24	103.58	6.46	98.61	5.91

### 3.6 Analysis of Real Patient Serum Samples

Human serum samples (*n* = 50) obtained from King Chulalongkorn Memorial Hospital were analyzed for CitH3 concentration using the proposed portable electrochemical immunosensor compared with those obtained from a standard ELISA method. A scatter plot of the correlation between CitH3 concentrations determined by the two methods yielded a fair correlation coefficient (*R*) of 0.9861 (Figure 7A). In addition, the agreement between the two methods was further verified using Bland–Altman analysis, as shown in Figure 7B. The differences between CitH3 concentrations determined by the two methods were within their mean ±1.96 SD, with 49 out of 50 samples falling within this range and only one outlier observed. This outlier corresponded to a particularly high concentration and did not significantly affect the overall interpretation. Thus, CitH3 determination results in real serum samples using the proposed immunosensor were in good agreement with those using the standard ELISA method.

**Figure 7:**
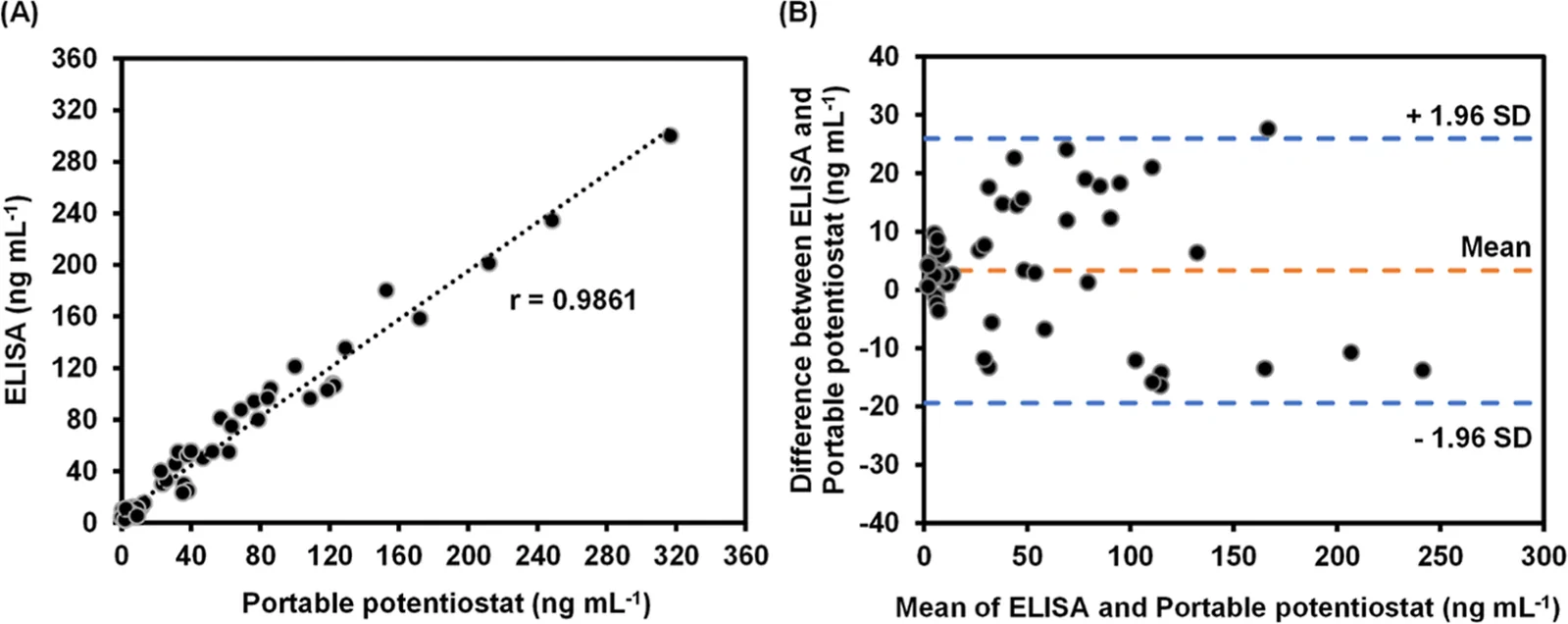
(A) Correlation analysis and (B) Bland–Altman agreement plot for the comparison of CitH3 concentrations measured by the developed electrochemical immunosensor and the ELISA method in human serum samples (*n* = 50).

The diagnostic performance of the developed assay was further analyzed in terms of its sensitivity and specificity for the identification of sepsis. Based on the calculation from the real data, the assay demonstrated a sensitivity of 90.01% and a specificity of 100% for distinguishing sepsis from nonsepsis patients. Additionally, the sensitivity and specificity for differentiating severe sepsis cases from nonsevere ones were 81.25% and 100%, respectively. These results demonstrated the potential of the developed assay for practical implementation in clinical settings. However, the present clinical validation was performed using samples from only a single medical center with a limited cohort size. Thus, further validation using multicenter clinical studies with more diverse patient populations would be required to confirm the robustness, generalizability, and clinical utility of the proposed platform before it is translated into routine clinical practices.

The present electrochemical immuno-sensing platform was compared with previously reported electrochemical systems for inflammatory and sepsis diagnosis as presented in Table 3. Apparently, other reported systems utilized quite complex electrode materials and different biomarkers, including CRP, IL-6, and PCT, for inflammatory and sepsis diagnosis. Some of these systems offered lower LODs and shorter assay times, but most of them were not portable due to the use of laboratory-based instrumentation and were not clinically validated. Furthermore, those biomarkers were not specific to sepsis, as their concentrations could be elevated under noninfectious inflammatory conditions.^8–13^ Thus, clinical identification of sepsis based on these markers would need additional data depending on patient conditions. In contrast, CitH3 offered high diagnostic specificity for sepsis associated with NET formation. Therefore, the developed SPGE-based portable device platform could be more promising as a practical low-cost POC platform for reliable sepsis diagnosis.

**Table 3:** Comparison of Previously Reported Electrochemical Systems for Inflammatory and Sepsis Diagnosis and the Present Electrochemical Immuno-Sensing Platform^a^

electrode	biomarker	LOD	assay time (min)	portability	clinical validation	references
aptamer/SPCE	CRP	54 ng mL^–1^	45	no	no	^35^
AuNPs/SPGE	CRP	1.5 ng mL^–1^	90	no	no	^36^
MWCNT/PCBG	CRP	745 ng mL^–1^	30	yes	yes	^37^
MXene/chitosan/PB/AuNPs modified SPCE	CRP	75 ng mL^–1^	30	no	no	^38^
	IL-6	7 pg mL^–1^	40	no	no	
P(o-PD)/SPCE	IL-6	1.74 pg mL^–1^	120	no	no	^39^
ZnO/gold electrode	PCT	0.1 μg mL^–1^	90	no	no	^40^
	CRP	0.1 ng mL^–1^	90			
AuNPs/GCE	PCT	0.33 pg mL^–1^	120	no	no	^41^
rGO-AuNPs/GCE	PCT	0.1 pg mL^–1^	120	no	yes	^42^
SPGE with a developed portable device	CitH3	42.83 pg mL^–1^	120	yes	yes	this study

a SPCE = screen-printed carbon electrode, AuNPs = gold nanoparticles, MWCNT = multiwalled carbon nanotubes, PCBG = printed circuit board gold, PB = Prussian blue, P­(o-PD) = poly­(*o*-phenylenediamine), rGO = reduced graphene oxide, ZnO = zinc oxide, and GCE = glassy carbon electrode.

## 4 Conclusion

In conclusion, a portable electrochemical immunosensor for CitH3 determination was successfully developed. The preparation parameters, including CitH3 antibody concentration, incubation time, BSA concentration, and BSA blocking time, were systematically optimized to attain high antibody surface coverage and minimal nonspecific binding. The enzymatic detection parameters—the time for CitH3 binding with immobilized antibodies, the concentration of HRP-conjugated antihistone H3 antibodies, and the time for HRP-conjugated antibodies binding with CitH3—were also optimized to obtain the best amperometric response and analytical sensitivity. Under optimal conditions, the device offered a wide linear range of 0.1–1000 ng mL^–1^ with a low LOD of 42.83 pg mL^–1^, allowing discrimination between clinically relevant CitH3 levels associated with sepsis and nonsepsis conditions. The analytical performance of the developed device was slightly inferior to that of a commercial potentiostat due to its relatively low bias stability, high background noise, and low measurement resolution. The potentiostat performance could be improved in the future by employing electromagnetic shielding on the device housing and implementing digital filtering algorithms on the microcontroller to reduce background noise and enhance signal resolution. Nevertheless, the platform gave advantages in portability, low cost, good reliability, short analysis time (∼2 h), and operational simplicity. Moreover, further reduction in assay time might be achieved through integration with agitation systems (e.g., orbital shaking or continuous flow) to improve mass transport. Also, the validated immunorecognition strategy could be adapted to lateral flow formats for future rapid POC diagnosis. Furthermore, correlation and Bland–Altman analyses of clinical results (*n* = 50) showed a good correlation coefficient (*r* = 0.9861) and good statistical agreement with the standard ELISA method, justifying its reliability. Lastly, the assay achieved decent diagnostic performance with high sensitivity values of 90.01% and 81.25% for identifying sepsis and severe sepsis cases, respectively (100% specificity for both cases). Therefore, the proposed immunosensor would be a potential practical POC testing tool for rapid sepsis diagnosis.

## References

[1] Cilloniz C., Martin-Loeches I., Garcia-Vidal C., San Jose A., Torres A. (2016). Microbial
Etiology of Pneumonia: Epidemiology, Diagnosis and Resistance Patterns. Int. J. Mol. Sci..

[2] Zandi M., Soltani S., Fani M., Abbasi S., Ebrahimi S., Ramezani A. (2021). Severe acute respiratory
syndrome coronavirus 2 and
respiratory syncytial virus coinfection in children. Osong Public Health Res. Perspect.

[3] Stupka J. E., Mortensen E. M., Anzueto A., Restrepo M. I. (2009). Community-acquired
pneumonia in elderly patients. Aging Health.

[4] Kang N., Subramanian V. S., Agrawal A. (2025). Influence of Aging and Immune Alterations
on Susceptibility to Pneumococcal Pneumonia in the Elderly. Pathogens.

[5] Li L., Hsu S. H., Gu X., Jiang S., Shang L., Sun G., Sun L., Zhang L., Wang C., Ren Y. (2020). Aetiology
and prognostic risk factors of mortality in patients with
pneumonia receiving glucocorticoids alone or glucocorticoids and other
immunosuppressants: a retrospective cohort study. BMJ. Open.

[6] V K., Mavani D. D., Patel D. V., Dantu G., Chaparala S. P., Song H. (2025). Mortality Related to
Pneumonia and Diabetes Mellitus: A Retrospective
Study. Cureus.

[7] Li S., Li L., Wang S., Wu H. (2025). Clinical characteristics and risk
factors of hospital mortality in elderly patients with community-acquired
pneumonia. Front. Med..

[8] Melbye H., Hvidsten D., Holm A., Nordbø S. A., Brox J. (2004). The course of C-reactive protein response in untreated upper respiratory
tract infection. Br. J. Gen. Pract..

[9] Christ-Crain M., Müller B. (2007). Biomarkers
in respiratory tract infections: diagnostic
guides to antibiotic prescription, prognostic markers and mediators. Eur. Respir. J..

[10] Kibe S., Adams K., Barlow G. (2011). Diagnostic and prognostic
biomarkers
of sepsis in critical care. J. Antimicrob. Chemother..

[11] Schuetz P., Albrich W., Mueller B. (2011). Procalcitonin
for diagnosis of infection
and guide to antibiotic decisions: past, present and future. BMC Med..

[12] Gomez B., Mintegi S., Bressan S., Da Dalt L., Gervaix A., Lacroix L. (2016). on behalf of the European
Group for Validation of the
Step-by-Step, A. Validation of the “Step-by-Step” Approach
in the Management of Young Febrile Infants. Pediatrics.

[13] Li H., Luo Y. F., Blackwell T. S., Xie C. M. (2011). Meta-analysis and
systematic review of procalcitonin-guided therapy in respiratory tract
infections. Antimicrob. Agents Chemother..

[14] Neeli I., Khan S. N., Radic M. (2008). Histone deimination
as a response
to inflammatory stimuli in neutrophils. J. Immunol..

[15] Papayannopoulos V. (2018). Neutrophil
extracellular traps in immunity and disease. Nat. Rev. Immunol..

[16] Wu Z., Deng Q., Pan B., Alam H. B., Tian Y., Bhatti U. F., Liu B., Mondal S., Thompson P. R., Li Y. (2020). Inhibition of PAD2 Improves Survival in a Mouse Model of Lethal LPS-Induced
Endotoxic Shock. Inflammation.

[17] Poli V., Zanoni I. (2023). Neutrophil intrinsic
and extrinsic regulation of NETosis
in health and disease. Trends Microbiol..

[18] Pan B., Alam H. B., Chong W., Mobley J., Liu B., Deng Q., Liang Y., Wang Y., Chen E., Wang T. (2017). CitH3:
a reliable blood biomarker for diagnosis and
treatment of endotoxic shock. Sci. Rep..

[19] Thålin C., Lundström S., Seignez C., Daleskog M., Lundström A., Henriksson P., Helleday T., Phillipson M., Wallén H., Demers M. (2018). Citrullinated histone H3 as a novel
prognostic blood marker in patients with advanced cancer. PLoS One.

[20] Mauracher L. M., Posch F., Martinod K., Grilz E., Däullary T., Hell L., Brostjan C., Zielinski C., Ay C., Wagner D. D. (2018). Citrullinated histone H3, a biomarker
of neutrophil extracellular trap formation, predicts the risk of venous
thromboembolism in cancer patients. J. Thromb.
Hemost..

[21] Grilz E., Mauracher L. M., Posch F., Königsbrügge O., Zöchbauer-Müller S., Marosi C., Lang I., Pabinger I., Ay C. (2019). Citrullinated
histone H3, a biomarker
for neutrophil extracellular trap formation, predicts the risk of
mortality in patients with cancer. Br. J. Hamaetol..

[22] Thålin C., Daleskog M., Göransson S. P., Schatzberg D., Lasselin J., Laska A. C., Kallner A., Helleday T., Wallén H., Demers M. (2017). Validation of an enzyme-linked
immunosorbent
assay for the quantification of citrullinated histone H3 as a marker
for neutrophil extracellular traps in human plasma. Immunol. Res..

[23] Tian Y., Russo R. M., Li Y., Karmakar M., Liu B., Puskarich M. A., Jones A. E., Stringer K. A., Standiford T. J., Alam H. B. (2021). Serum citrullinated histone H3 concentrations differentiate
patients with septic verses non-septic shock and correlate with disease
severity. Infection.

[24] Primpray V., Kamsong W., Pakapongpan S., Phochakum K., Kaewchaem A., Sappat A., Wisitsoraat A., Lomas T., Tuantranont A., Karuwan C. (2022). An alternative ready-to-use
electrochemical immunosensor for point-of-care COVID-19 diagnosis
using graphene screen-printed electrodes coupled with a 3D-printed
portable potentiostat. Talanta Open.

[25] Permpoka K., Kaewarsa P., Paekoh W., Opanuraks J., Laiwattanapaisal W., Estrela P. (2025). A capacitance biosensor for prostate
cancer detection via normalised urinary extracellular vesicles. Biosens. Bioelectron..

[26] Rabbani G., Khan M. E., Zakri W., Khan M. V., Bashiri A. H. (2024). An electrochemical
immunosensor based on AgNPs/Nafion-GCE for detection of salivary lactoferrin:
Alzheimer’s disease biomarker. Microchem.
J..

[27] Kucuk I., Sadak S., Kurnalı S. Z., Altınöz S., Uslu B. (2025). A novel electrochemical sensor based
on MIP technology for sensitive
determination of cinacalcet hydrochloride in tablet dosage form and
serum samples. Microchim. Acta.

[28] Zvirzdine G., Zukauskas S., Rucinskiene A., Mohsenzadeh E., Boguzaite R., Ramanaviciene A., Pogorielov M., Ratautaite V., Ramanavicius A. (2025). Electrochemical
Salicylic Acid Sensor
Based on Molecularly Imprinted Polypyrrole. ACS Appl. Mater. Interfaces.

[29] Chen C., Xia P., Chen M., Dong W., Bai R., Shan L., Feng S. (2026). A Sequential Epitope-Protein Imprinting Strategy for Antibody-Free
Electrochemical Sensing of CD44 Glycoprotein with Ultralow Detection
Limit. Anal. Chem..

[30] Allen M. J., Tung V. C., Kaner R. B. (2010). Honeycomb
Carbon: A Review of Graphene. Chem. Rev..

[31] Zhang X., Cao S., Wu X., Hu L. (2025). Overview on Comparison of Four Preparation
Methods and Physical Properties of Graphene. Nanomater. Nanotechnol..

[32] Ren H. (2025). Graphene and
Its Derivatives for Electrochemical Sensing. Sensors.

[33] Singer M., Deutschman C. S., Seymour C. W., Shankar-Hari M., Annane D., Bauer M., Bellomo R., Bernard G. R., Chiche J. D., Coopersmith C. M. (2016). The Third International
Consensus Definitions for Sepsis and Septic Shock (Sepsis-3). Jama.

[34] Dong H., Li C. M., Chen W., Zhou Q., Zeng Z. X., Luong J. H. T. (2006). Sensitive Amperometric
Immunosensing Using Polypyrrolepropylic
Acid Films for Biomolecule Immobilization. Anal.
Chem..

[35] Centi S., Bonel Sanmartin L., Tombelli S., Palchetti I., Mascini M. (2009). Detection of C Reactive
Protein (CRP) in Serum by an
Electrochemical Aptamer-Based Sandwich Assay. Electroanalysis.

[36] Jampasa S., Siangproh W., Laocharoensuk R., Vilaivan T., Chailapakul O. (2018). Electrochemical
detection of c-reactive protein based on anthraquinone-labeled antibody
using a screen-printed graphene electrode. Talanta.

[37] Zambry N. S., Prathaban S. N., Ibrahim F., Thiha A., Karajacob A. S., Jamaluddin N. F., Omar S. F. S., Shariffuddin I. I., Bustam A., Tay S. T. (2025). A label-free electrochemical immunosensor
on gold-printed electrode for the electrochemical detection of C-reactive
protein from blood samples. Microchim. Acta.

[38] Shi Z., Li K., Wang Y., Li Z., Zhu Z., Wang L. (2025). Development
of a dual-channel electrochemical aptasensor for simultaneous detection
of CRP and IL-6 in sepsis diagnosis. Electrochim.
Acta.

[39] Ting W.-T., Wang M.-J., Howlader M. M. R. (2024). Interleukin-6
electrochemical sensor
using poly­(o-phenylenediamine)-based molecularly imprinted polymer. Sens. Actuators, B.

[40] Tanak A. S., Jagannath B., Tamrakar Y., Muthukumar S., Prasad S. (2019). Non-faradaic electrochemical
impedimetric profiling
of procalcitonin and C-reactive protein as a dual marker biosensor
for early sepsis detection. Anal. Chim. Acta:X.

[41] Zhao G., Yan Q., Wang B., Wang N., Duolihong B., Xia X. (2022). CoFe-(oxy)­hydroxide as a novel electrocatalytic
tag in immunosensing
for ultra-sensitive detection of procalcitonin based on the oxygen
evolution reaction. Bioelectrochemistry.

[42] Liu P., Li C., Zhang R., Tang Q., Wei J., Lu Y., Shen P. (2019). An ultrasensitive
electrochemical immunosensor for procalcitonin
detection based on the gold nanoparticles-enhanced tyramide signal
amplification strategy. Biosens. Bioelectron..

